# The Environmental Factors Associated With Fatigue of Frontline Nurses in the Infection Disease Nursing Unit

**DOI:** 10.3389/fpubh.2021.774553

**Published:** 2021-12-06

**Authors:** Ming Ma, Michael Adeney, Hao Long, Baojie He

**Affiliations:** ^1^School of Architecture and Urban Planning, Chongqing University, Chongqing, China; ^2^Key Laboratory of Technology for Construction of Cities in Mountain Area of Ministry of Education, Chongqing University, Chongqing, China; ^3^School of Public Health, University of Washington, Seattle, WA, United States

**Keywords:** nurse, infection disease nursing unit, fatigue, environmental design, COVID-19, hospital

## Abstract

The workload in the Infection Disease Nursing Unit (IDNU) is increasing dramatically due to COVID-19, and leads to the prevalence of fatigue among the frontline nurses, threatening their health, and safety. The built environment and design could fundamentally affect the fatigue of nurses for a long-term perspective. This article aims to extract the environmental factors of IDNU and explore nurses' perceptions of these factors on the work-related fatigue. It would produce evidences for mitigating the fatigue by environmental interferons. A cross-sectional design was employed by combination of focus group interview and written survey. Environmental factors of IDNU were collected from healthcare design experts (*n* = 8). Nurses (*n* = 64) with frontline COVID-19 experiences in IDNU were recruited to assess these factors individually. Four environmental factors were identified as: Nursing Distance (ND), Spatial Crowdness (SC), Natural Ventilation, and Light (NVL), and Spatial Privacy (SP). Among them, ND was considered as the most influential factor on the physical fatigue, while SP was on the psychological fatigue. Generally, these environmental factors were found to be more influential on the physical fatigue than the psychological fatigue. Technical titles were found to be associated with the nurses' perceptions of fatigue by these environmental factors. Nurse assistant and practical nurse were more likely to suffer from the physical fatigue by these factors than senior nurse. The result indicated that environmental factors of IDNU were associated with the nurses' fatigue, particularly on the physical aspect. Environmental interventions of design could be adopted to alleviate the fatigue by these factors such as reducing the ND and improving the spatial privacy. The accurate interventional measures should be applied to fit nurses' conditions due to their technical titles. More attention should be given to the low-ranking nurses, who account for the majority and are much vulnerable to the physical fatigue by environmental factors.

## Introduction

Fatigue refers to a sub-health state without specific symptoms, such as psychological and physical fatigue (1). Work-related fatigue harms the efficiency, health, and safety of the nurses by producing symptoms, such as headache, dizziness, anxiety, depression, compulsion, and insomnia (2–4). It could also increase the risk of injures and medication errors of nurses, which are compounded by poorly designed working space and environment (5–7). The anxiety feelings and burnout due to lasting fatigue could reduce nurses' job satisfaction and encourage the change of occupations (8). The studies revealed that the overgrowing shortages of nurses were closely associated with the exposure to a high degree of work-related fatigue (9–11).

The breakout of COVID-19 has further exacerbated the prevalence of fatigue among nurses (12). The rapid accumulation of confirmed cases and the fear of infections have been increasing the chance of fatigue (13). The workload of nurses is increasing dramatically for medication, testing, prevention, and quarantine. In some countries, the work hours of nurses have been increased by 1.5–2 times on average, which fundamentally affects their perceptions of the work environment (14). The overwhelming workload does not only produce physical exhaustion, but also increase the psychological burden (15). During COVID-19, nurses usually experienced depression, exhausted stress, insomnia, and other negative emotions (2, 3). The long-term exposure to medication procedures such as intubation and nebulization also leads to the high percentage of fatigue by fear of the hospital-acquired infections (16, 17). Therefore, reducing the fatigue by tailored measures is critical to promote nurses' safety and health (18), and the environmental interventions could become a promising approach.

There are plenty of sources for the fatigue of nurses such as professional pressure, heavy workload, working environment, and patient care (19–21). COVID-19 makes the situation even complicated due to the fear of infection and the change of work conditions. A study in Australia summarized the emerging sources as: patient care, changed work conditions, isolation and uncertainty, and lack of support (22). The concerns of infection spreading to families, and community would exacerbate the issue (23). In the beginning of pandemic, the shortage of PPE (Personal Protection Equipment) and moral dilemmas also added an extra burden to escalate the fatigue (17, 24, 25). The lessons from COVID-19 showed that the existing working environment cannot fully meet nurses' physical and psychological conditions in the pandemic. Except for management and counseling, promoting the physical environment could also alleviate the fatigue of nurses (26, 27). It comprises a cost-effective intervention for creating safe and healthy environments for occupants in the hospital and is gaining increasing attention (28).

The physical design of the healthcare environment is important to the fatigue of the medical staffs by providing healthy and safe working condition (11). The environmental factors affecting fatigue could range from spatial side like plan, layout, and room setting, to the physical side such like noise, light, and air pollution (9, 14, 26, 29, 30). Among the physical factors, the noise was reported to be closely associated with the mental and psychological fatigue of the staffs by interfering with the working environment. High noise level could affect the staffs' comfort and satisfaction, leading to the lasting status of fatigue (7, 27, 31). Natural light was believed to mitigate fatigue by reducing the stress and anxiety of the nurses (27, 29). In a healthcare setting, natural light, fresh air, and views on of green space could effectively reduce the psychological fatigue of the patients and staffs due to restoration of nature, in both visual and physical contact (9, 26, 32). Introducing daylighting by arranging window toward natural and green resources could reduce the fatigue and stress of the occupants by enhancing their satisfaction, mood, and alertness (33, 34). Though the knowledge from a spatial perspective was limited, some studies still revealed that environmental factors such as functional layout, interior exterior, and environmental features were associated with the fatigue in the various setting (27, 29). These factors could alleviate the stress, emotional exhaustion, and depression of staff once the environment fits their physical and psychological needs (26, 35). The layout of rooms could affect the medical staff' fatigue by interfering their behaviors of moving, resting, and working. The impact occurs throughout the entire process of workflow, such as medication ordering, storage, delivery, dispensation, preparation, and administration (22). Creating break and communication areas for nurses could help build up a supportive environment for alleviating the fatigue (36, 37). A short break was reported to enhance the mental and psychological wellbeing of staff, as well as reduce their fatigue (27). This dynamic could be magnified by improving the restorative quality of break areas *via* environmental design, leading to the substantially increasing of the staff's satisfaction and the mitigation of fatigue. Some studies also implied the importance to connect the interior space to the natural resources in a healthcare setting. The access to green space both visually and physically was reported to alleviate relief staff fatigue effectively by increasing the “awareness,” “accessibility,” and “comfort” of nature (38). Except for the spatial factors, detailed features of the healthcare environment are also associated with the fatigue. Flooring that provides comfort, easy cleanability, and sound absorption could mitigate the staff's fatigue by affecting their perceptions of working environment (39, 40). Water features, in either of natural and artificial way, were believed to reduce stress and anxiety of the medical staff (41, 42). Overall, the current studies indicated the importance of environmental factors to medical staff' fatigue, with emphasis on the physical aspect. Nonetheless, the knowledge is limited on the specific healthcare setting with the certain group of occupants. Some workplace settings were reported to produce more fatigue than others, which indicated the necessity to dig into the specific healthcare setting in COVID-19 (43). Moreover, the findings were often general and the principles used in the practice often simply followed the codes and office buildings (44), while there were fewer studies on the spatial aspect of the environment than the physical.

In COVID-19, the Infection Disease Nursing Unit (IDNU) is playing an important role in treating the confirmed cases, and the nurses who work there are often vulnerable to the fatigue due to the heavy workload and exposure to the pathogen. To the best of our knowledge, there are few studies on the frontline nurses and the environmental setting in IDNU. To fill the knowledge gap, the exploratory study aims to extract and identify the main environmental factors of IDNU affecting the fatigue from perspective of design. The focus group interview and survey were employed to explore the environment of IDNU and fatigue, from healthcare design experts and frontline nurses, respectively. The article would provide evidence to build up a safe and healthy nursing unit during and after the pandemic.

## Methods

**Figure 1:**
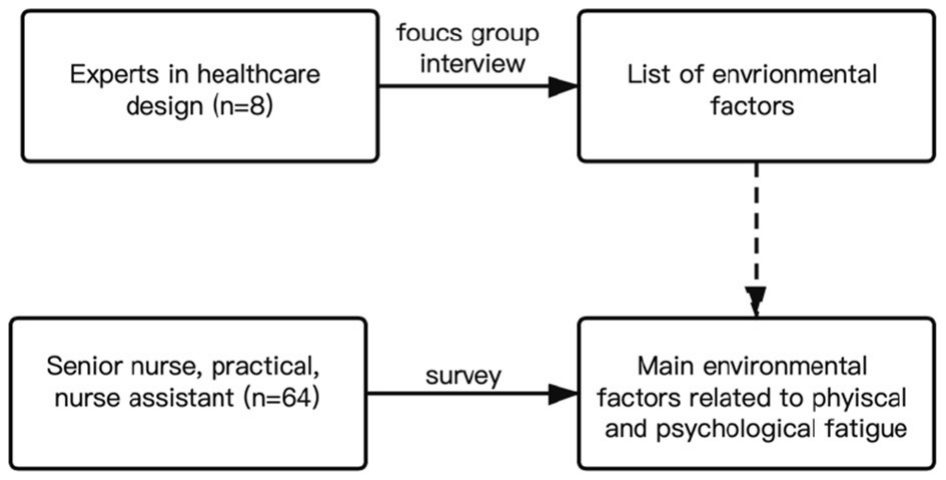
Conceptual research framework.

This study employed a combination of focus group interview and written survey, on the experts and nurses respectively, for the data of environmental factors and fatigue. It allowed for the triangulation of findings and being easy to draw definite conclusions (Figure 1). For the definition of “fatigue,” it referred to a “work-related condition that ranges from acute to chronic in nature and can result in an over-whelming sense of tiredness, decreased energy, and exhaustion, ultimately accompanied by impacting physical and cognitive functions” (20). For the environmental factors, they referred to these of environmental design, including architectural design and interior design, such as the floor plan, functional layout, room setting, ventilation, and lighting.

### Focus Group Interview

This method was applied to collect experts' thoughts to extract environmental factors because it was effective on the qualitative information of contextualized and complicated content. Eight experts with healthcare design backgrounds and IDNU experiences joined the interview and provided the information and opinions as to the environmental factors. Their detailed perspectives on the various topics were also explored to provide a holistic comprehension. The group size was not large in order to enable enough in-depth discussion. This approach would enable the experts to provide comprehensive information of environmental factors based on a specific case. The results of the interview were analyzed by two investigators and then synthesized into a list of environmental factors, which constituted the basis for the written survey.

The focus group interview followed three topics prepared by interviewers which comprised of: (1) The environmental factors and intervention in the IDNU from design. (2) The role of environmental design in affecting nurses in COVID-19. (3) The promising design measures and strategies to reduce the fatigue. For each topic, we prepared a list of discussion questions in order to guide the experts round the topics. The interviews were recorded, analyzed, and coded by the other two investigators. All the investigators and interviewers were trained to ensure the quality of interview. The answers and discussion of topics were divided into sub-topics, interpreted, compared, and sorted out. The environmental factors were then categorized by counting the frequencies of experts' agreements on them.

### Written Survey

A written survey was adopted to collect the nurses' perceptions of environmental factors on the fatigue. The survey was conducted in the form of online questionnaires due to the restrictions in the pandemic. These questionnaires were based on the results of the focus group interview and designed as semi-structural. The questionnaires consisted of three parts (Appendix A): The first part was about their social-demographic information on gender, age, length of practice, titles, and education level (Table 1). The second part was the rating of the environmental factors regarding its importance on their psychological and physical fatigue (Likert approach, 1–5). The third part was the locations in the IDNU where nurses often worked and stayed. The questionnaires were distributed in the Wechat group of the qualified nurses, and they could answer the survey on their worksites in a given time. The questionnaires were developed with the tool of Questionnaire Star, which was a plug-in based on the Wechat platform (https://www.wjx.cn). This method followed the regulations of the hospital and could reduce the disturbance of the nurses of IDNU. The main environmental factors on the fatigue would be determined based on the results of the survey. The questionnaires were designed and reviewed without the violation of personal privacy. They were anonymous, and the participants were allowed to terminate the survey any time they desired. The focus group interview was conducted from 15 to 18 October, 2020.

**Table 1:** Characteristics of the participants.

	**Frontline Nurses**			**Healthcare Experts**	
		**Number**	**Percent**	**Number**	**Percent**
Age	<30	42	65.6%	0	0.0%
	30–40	18	28.1%	2	25.0%
	40–60	4	6.3%	6	75.0%
Gender	Male	2	3.1%	7	87.5%
	Female	62	96.9%	1	12.5%
Technical title	Nurse assistant	24	37.5%	–	–
	Practical nurse	32	50.0%	–	–
	Senior nurse	8	12.5%	–	–
Work duration in COVID-19	>25 days	15	23.4%	–	–
	<25 days	49	76.6%	–	–
Education	Undergraduate	21	32.8%	–	–
	Graduate	43	67.2%	–	–
Length of profession	1–4 years	13	20.3%	0	0.0%
	5–9 years	42	65.6%	0	0.0%
	>10 years	9	14.1%	8	100.0%

### Participants and Site

In the focus group interview, the participants were consisted of eight healthcare design experts who were recruited from the Shenzhen Architectural Design Group, which was also the design contractor of the targeted IDNU. They were recruited through the team's extensive professional network and joined the interview voluntarily. The recruiting criteria were: (1) They were required to have experiences of three IDNU projects at least. (2) They were required to have professional experiences for at least 10 years in the healthcare sector. (3) They should fully understand the purpose and expected results of the interview.

The nurses of the written survey were recruited from the department of IDNU in Shenzhen 3rd People Hospital. In the pandemic, it was impossible to initiate the massive survey to the nurses in the IDNU due to restrictions on the contact with them. The number of nurses in the IDNU was also much less that of other nursing units. Hence, we decided to focus on one typical and large IDNU to ensure the quality of the survey. We distributed the requirements of the survey to all the nurses in that department with the assistance of hospitals. They were all trained and managed in the similar system with these from other public hospitals, which accounted for approximately 3/4 of all in-patient beds nationwide. The selection criteria included: (1) They had to work full-time in the IDNU; (2) They had the frontline experiences with COVID-19 cases, which meant they engaged in the direct treatment and care of confirmed cases; (3) They worked in the IDNU for a year at least. Qualified respondents would be identified as volunteer participants and invited in the survey group, after obtaining their verbal consents. Eventually, we received 64 valid feedbacks out of 72 qualified nurses. The written survey was conducted from 11 to 30 November, 2020.

**Figure 2:**
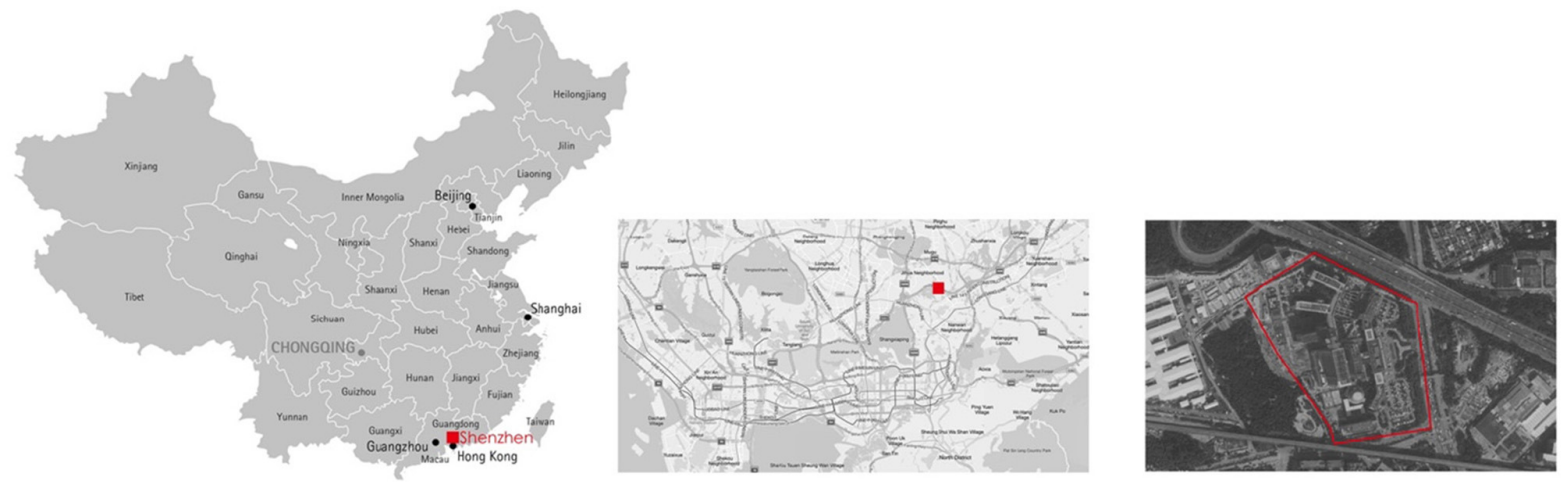
The location of the Shenzhen Third People Hospital.

**Figure 3:**
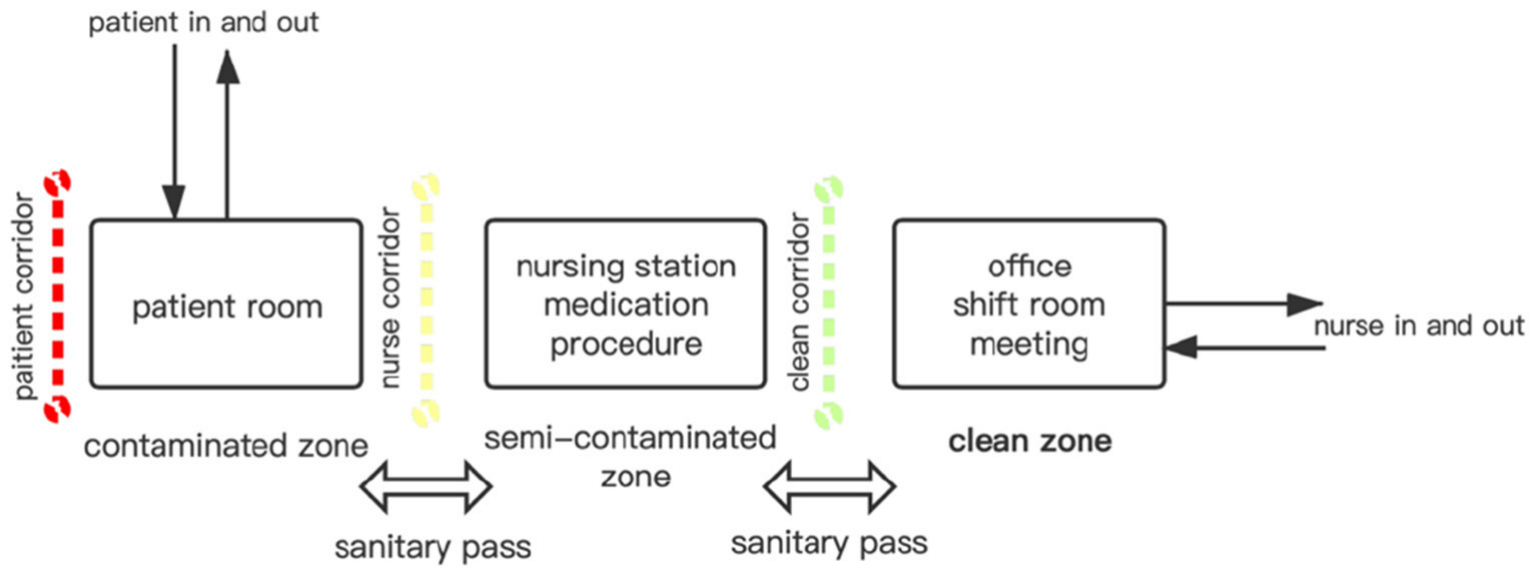
The “Three Zone and Three Corridors” mode of infection disease nursing infection disease nursing unit.

**Figure 4:**
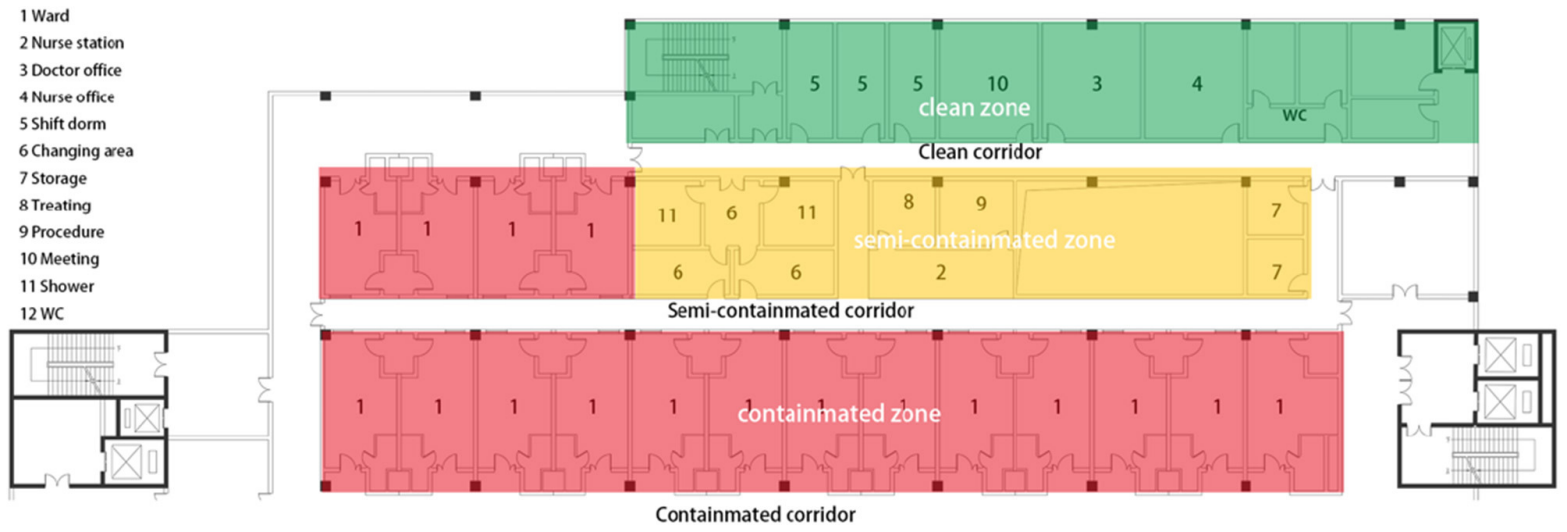
Plan of IDNU in the Shezhen 3rd People Hospital.

The IDNU of Shenzhen 3rd People Hospital was selected in this study because it was the only designated COVID-19 treatment hospital in Shenzhen for its capacity and specialty (Figure 2). In the pandemic, the administration requested all the moderate-to-critical-condition COVID-19 cases to be transferred to this hospital for medication. The environmental design of that IDNU is very representative in China, which is featured with “three zones and three corridors” (Figure 3). As a special type of nursing unit, IDNU is more complicated than normal ones because it was designed to accommodate highly contagious patients and followed rather rigorous infection control protocol (Figure 4). Since all the IDNU were owned by the public hospitals, they were all designed and built under the same codes and guidelines.

### Data Analysis

Expect for conventional descriptive analysis, one-way ANOVA, and *t*-test were used to compare the environmental factors on the physical and psychological fatigue respectively, by age, gender, technical titles, and education. To find out the correlations of these factors on the fatigue, Pearson's correlation analysis was performed. The reliability of questionnaires was tested with the Cronbach's coefficient which was 0.792, indicating good reliability. The validity of the data was tested by KMO and Bartlett test and proved to be suitable of research with KMO of 0.771. The statistical analysis was conducted on the SPSS V26.0 (IBM Corp, Armonk, NY, USA). The significance level was set at *p* = 0.05.

## Results

### Environmental Factors of IDNU

According to the focus group interview, the environmental factors of IDNU could be sorted into four main categories which are Nursing Distance (ND), Spatial Crowdness (SC), Natural Ventilation, and Light (NVL), and Spatial Privacy (SP) (Table 2). By counting the agreements of experts, ND was thought to be the most important factor with total counts of 21. It would affect how much distance nurses would walk every day, which was considered as a prominent indicator of physical exhaustion (37). It could be determined by the plan layout (8), location of nursing station (6), and number of wards (7). In this case, the plan layout of IDNU was linear and featured with “three zone and three corridors,” which was the most common type of IDNU. The nurse station was located in the middle of the nursing unit. SC received total counts of 11. It could affect the nurses' perceptions of crowdness by spatial layout and design (30). The factors were consisted of size of nurse station (3), ratio of supportive rooms (4), and width of corridor (4). In this IDNU, the size of nurse station was 2.4 m × 7.2 m (depth and length), which was standard on every IDNU floor. The proportion of supportive rooms (rooms except for wards and medical rooms) was 32.2%, indicating a compact layout. The width of the main corridor was 2.4 m in this case. Natural light and ventilation were identified with the total counts of 12. It was reported to be associated with the stress and anxiety reduction (38), and mainly determined by the arrangements and design of windows. It included the factors of daylight around the nursing station (5), openable windows in the end of corridor and break area. In this case, there were openable windows at the end of corridor. The last factor was SP that received the total counts of 17, just behind the nursing distance. It referred to the perceptions of privacy in a spatial setting, and could be profoundly affected by environmental design. It included the sub-factors of independent break areas (8), safety of workplace (6), and typology of nurse (3). In this IDNU, there were no independent break areas for nurses. The access of their workplace was restricted to the visitors but connected to the patients. Their nurse station was open to the semi-contaminated area, where the exposure to the infection was high.

**Table 2:** The environmental factors relevant to fatigue in infection disease nursing unit.

**Main Categories**	**Sub Categories**	**Descriptions in the IDNU**
A Nursing Distance (21)	A1 Plan layout (8) A2 Location of nurse station (6) A3 Number of wards (7)	A1 Linear + “three zone and three corridors” A2 Middle A3 17
B Spatial Crowdness (11)	B1 Size of nurse station (3) B2 Ratio of supportive rooms (4) B3 Width of corridor (4)	B1 2.4 * 7.2m B2 32.2%(area) B3 2.4m
C Natural ventilation and Light (NVL) (12)	C1 If there is daylighting in the nurse station (5) C2 If openable windows in the end of corridor (2) C3 If access to natural light and ventilation in the break area (5)	C1 no C2 Yes C3 no
D Spatial Privacy (SP) (17)	D1 Independent break area for nurse (8) D2 Safety of workplace (6) D3 Typology of nurse station(open/semi-open/closed)	D1 no D2 n/a D3 open

*The scores were calculated based on the frequencies of experts' agreements on the environmental factors*.

### Nurse Response on the Environmental Factors

**Figure 5:**
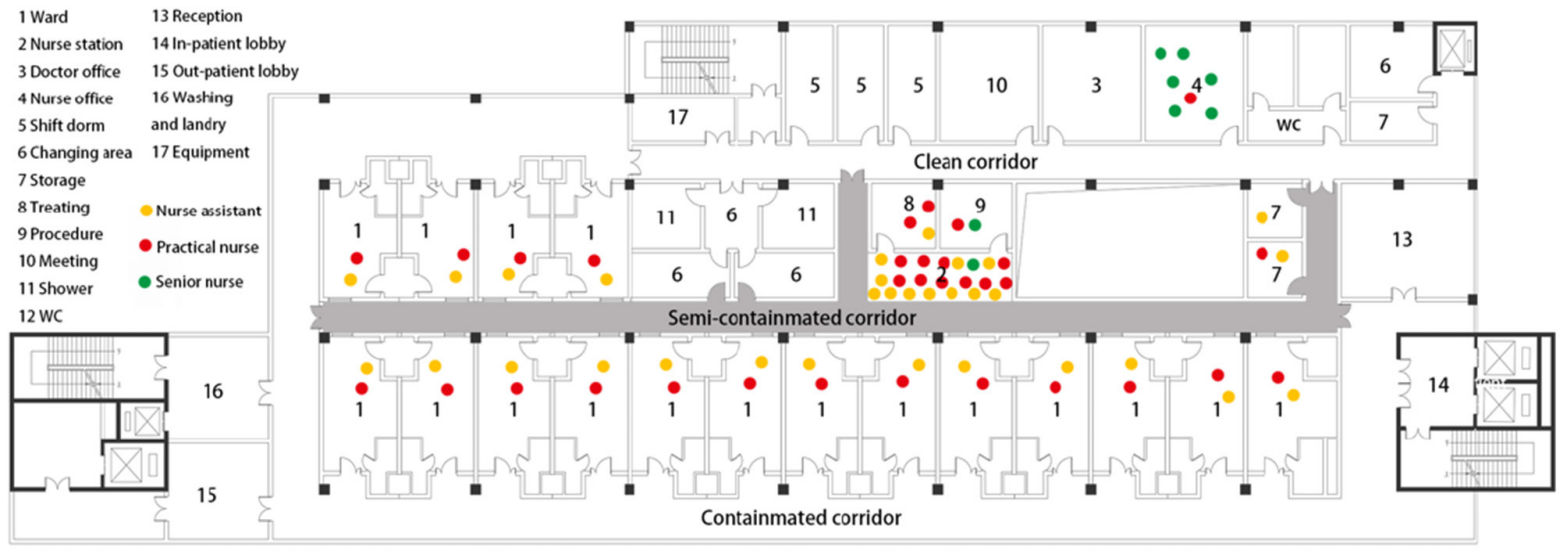
The locations of nurses frequently stay and work.

The environmental factors were rated by the nurses to determine their influence on the fatigue. The mean value of scores on the physical fatigue was higher than that on the psychological fatigue generally, indicating that these factors could significantly influence the physical fatigue (Table 3). Among them, ND was rated the highest on physical fatigue, with a mean value of (3.92), while SP was rated the highest (3.55) on the psychological fatigue. Regarding the frequent locations of nurses, it was apparent that senior nurses were mostly staying in the clean and semi-contaminated zone, while nurse assistants and practical nurses were often in the semi-contaminated zone and contaminated zone with frequent commuting from wards to nursing station (Figure 5). One-way ANOVA was conducted among clusters of nurses to compare these factors on the physical or psychological fatigue. The approximate normal distributions and equal variances were tested and satisfied by Shapiro–Wilk test of normality and Levene's test for homogeneity. The results revealed that technical titles could affect the nurses' perceptions of the fatigue from the environmental factors. The ratings from senior nurses were lower than practical nurses and nurse assistants, indicating that environment had less influence on the senior nurses than others, and were more evident on the low-ranking nurses.

**Table 3:** The descriptions of the environmental factors on the fatigue.

		**ND**		**SC**		**NVL**		**SP**	
		**Physical**	**Psychological**	**Physical**	**Psychological**	**Physical**	**Psychological**	**Physical**	**Psychological**
		**fatigue**	**fatigue**	**fatigue**	**fatigue**	**fatigue**	**fatigue**	**fatigue**	**fatigue**
		**M (SD)**	**M (SD)**	**M (SD)**	**M (SD)**	**M (SD)**	**M (SD)**	**M (SD)**	**M (SD)**
General	Mean	3.92 (1.12)	3.21 (1.43)	2.33 (0.96)	2.70 (0.81)	2.24 (1.01)	2.52 (0.98)	3.33 (1.31)	3.55 (1.14)
Senior nurse	Mean	3.14 (0.71)	3.51 (1.12)	2.11 (1.27)	3.14 (1.07)	1.81 (1.05)	2.71 (0.99)	2.91 (1.34)	3.91 (1.13)
Practical nurse	Mean	3.91 (0.94)	3.15 (0.95)	2.33 (1.15)	2.54 (0.99)	2.24 (1.14)	1.92 (0.97)	3.54 (0.95)	3.34 (1.21)
Nurse assistant	Mean	4.12 (1.10)	3.57 (1.03)	2.71 (1.05)	2.42 (0.96)	2.64 (1.12)	2.93 (1.05)	3.65 (0.93)	3.23 (1.18)

*Total N = 64, M = Mean, SD = Standard Deviation*.

*ND, Nursing Distance; SC, Spatial Crowdness; NVL, Natural Ventilation and Light; SP, Spatial Privacy*.

#### Environmental Factors on Physical Fatigue

As for the physical fatigue, nurses collectively rated the highest on the ND with a mean value of 3.92 (Table 3), followed by SP (3.33). NVL was rated the lowest (2.24), then following by SC (2.33). SP was rated with a mean value of (3.33). The results implied that ND and SP were more important on physical fatigue than the other two. According to the Pearson's correlation analysis, ND was positively associated with SP (*r* = 0.275, *p* < 0.000) while negatively associated with SC (*r* = −0.230, *p* < 0.000) (Table 4). Hence, ND could be identified as the main environmental factor that affects the physical fatigue.

**Table 4:** Correlations of environmental factors on the physical fatigue.

**Variables**		**ND**	**SC**	**SP**	**NVL**
ND	*r*	1.000	−0.230	0.275	0.081
	*p*	–	0.000	0.000	0.106
SC	*r*	−0.230	1.000	−0.064	−0.064
	*p*	0.000	–	0.180	0.180
SP	*r*	0.275	−0.064	1.000	−0.012
	*p*	0.000	0.180	–	0.838
NVL	*r*	0.081	0.064	−0.012	1.000
	*p*	0.106	0.180	0.838	–

*Correlation is significant at the 0.05 level (2-tailed)*.

**Figure 6:**
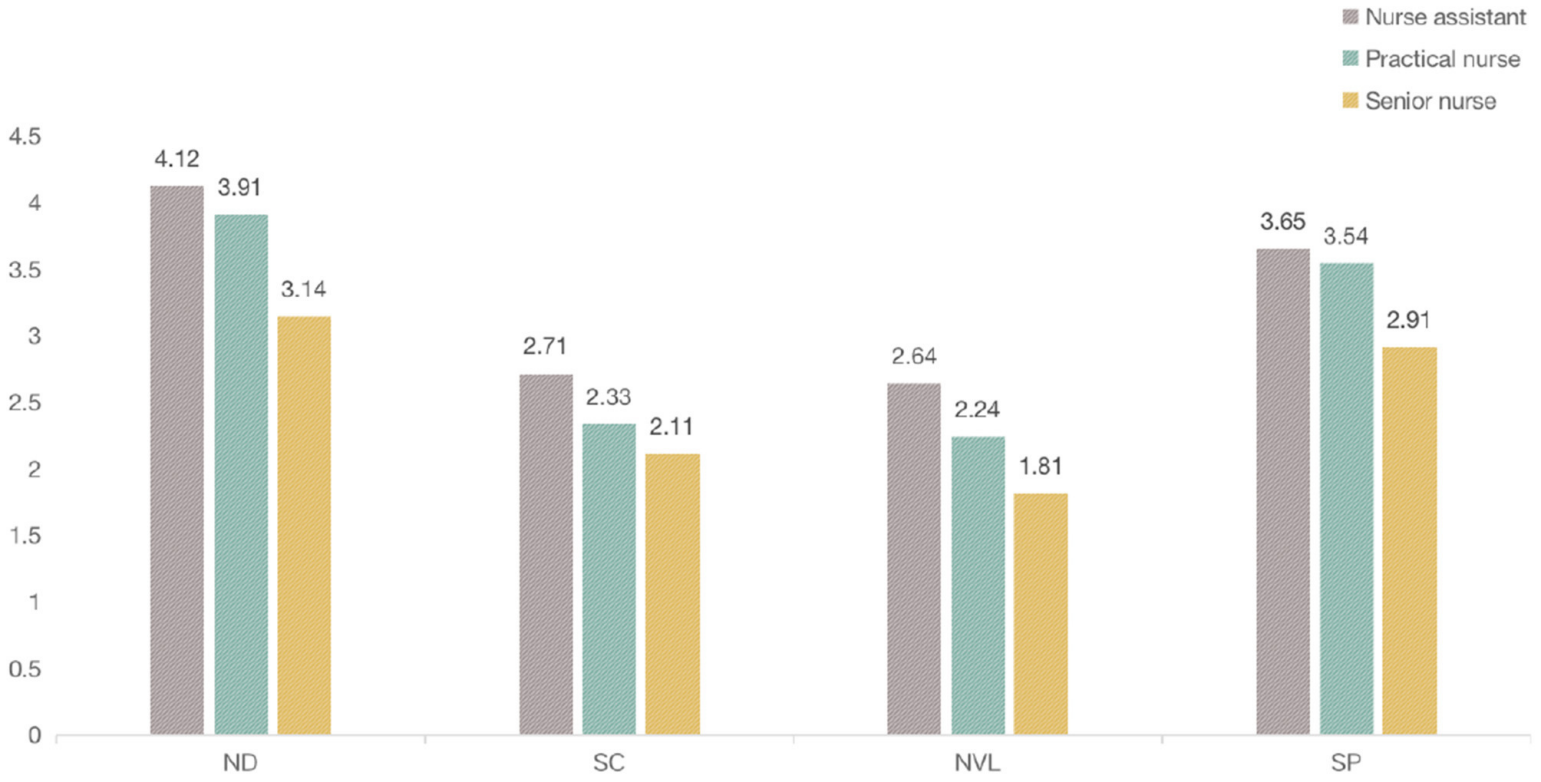
Importance of environmental factors on psychological fatigue.

The technical titles of nurses showed significant differences among ND (*F* = 2.142, *p* = 0.034), NVL (*F* = 2.372, *p* = 0.031), SP (*F* = −1.97, *p* = 0.041). Work duration in COVID-19 showed statistical differences among ND (*F* = −3.295, *p* = 0.001). By comparing the mean value, it was found that nurse assistants rated higher than practical nurses, and the senior nurses were indicating that low-ranking nurses were more likely to be influenced by these factors on the physical fatigue than the senior nurses (Figure 6).

#### Environmental Factors on Psychological Fatigue

**Figure 7:**
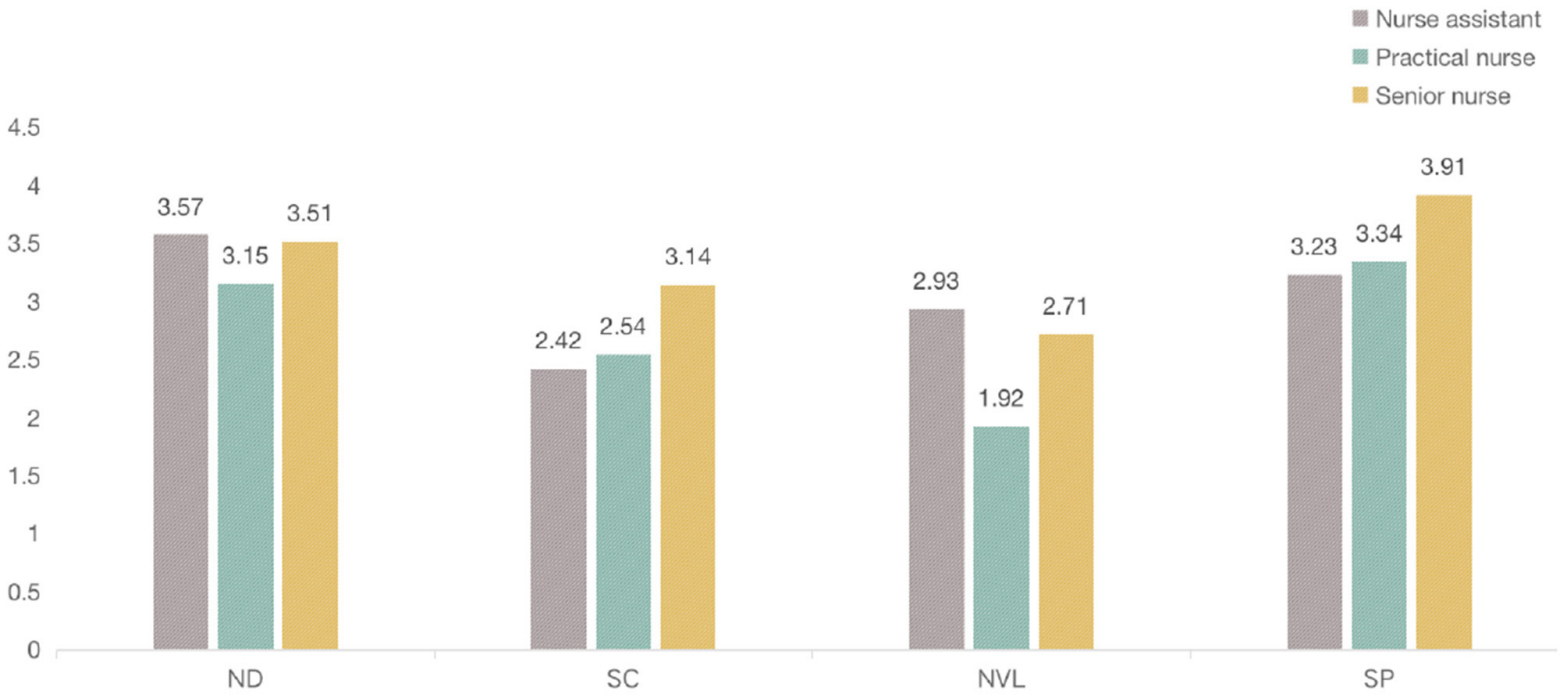
Importance of environmental factors on the psychological fatigue.

Spatial privacy was rated the highest mean value (3.55), followed by ND (3.21) (Figure 7). Similar with physical fatigue, NVL was rated the lowest (2.52), which was a little lower than SC (2.70). According to the Pearson's correlation analysis, significant associations were only found between SP and the other three factors (Table 5). It revealed that SP was supposed to be the primary environmental factor.

**Table 5:** Correlations between environmental factors on the psychological fatigue.

**Variables**		**ND**	**SC**	**SP**	**NVL**
ND	*r*	1.000	0.099	0.371	0.100
	*p*	–	0.117	0.000	0.095
SC	*r*	0.099	1.000	0.224	0.078
	*p*	0.117	–	0.000	0.087
SP	*r*	0.371	0.224	1.000	0.138
	*p*	0.000	0.000	–	0.021
NVL	*r*	0.100	0.078	0.138	1.000
	*p*	0.095	0.087	0.021	–

*Correlation is significant at the 0.05 level (2-tailed)*.

As observed in ANOVA analysis, technical titles had significant differences among ND (*F* = 4.276, *p* = 0.007), NVL (*F* = −1.896, *p* = 0.031), and SP (*F* = −2.89, *p* = 0.004), regarding the psychological fatigue. Working duration in the COVID-19 showed a significant difference within SC (*F* = 2.456, *p* = 0.021) (Table 6).

**Table 6:** Social-demographic characteristics by the environmental factors.

**Factor**	**Physical fatigue**							
	**ND**		**SC**		**NVL**		**SP**	
	***T*/*F* value**	***P*-value**	***T*/*F* value**	***P*-value**	***T*/*F* value**	***P*-value**	***T*/*F* value**	***P*-value**
Age	0.069	0.945	2.616	0.078	1.539	0.219	1.1	0.347
Gender	1.195	0.234	–0.946	0.346	0.237	0.813	1.65	0.099
Technical title	2.142	0.034^*^	−1.084	0.281	2.372	0.031^*^	−1.97	0.041^*^
Work duration in COVID-19	-3.295	0.001^*^	−0.195	0.846	0.332	0.741	–0.83	0.406
Education	0.17	0.866	–1.003	0.318	–0.651	0.516	1.15	0.271
Length of profession	0.066	0.936	–1.084	0.281	–0.139	0.89	1.88	0.076
	**Psychological fatigue**							
Age	0.17	0.866	–1.575	0.118	0.806	0.422	0.38	0.764
Gender	0.554	0.581	0.873	0.384	0.753	0.453	0.35	0.727
Technical title	4.276	0.007^*^	1.131	0.311	−1.896	0.031^*^	−2.89	0.004^*^
Work duration in COVID-19	–1.654	0.101	2.456	0.021^*^	0.215	0.831	–0.35	0.729
Education	1.822	0.071	1.245	0.297	2.057	0.110	0.611	0.539
Length of profession	–1.266	0.208	–0.295	0.769	0.765	0.446	0.092	0.913

*T, value for independent-samples t-test; F, F ratio for ANOVA*

* *p < 0.05; ND, Nursing Distance; SC, Spatial Crowdness; NVL, Natural Ventilation and Light; SP, Spatial Privacy*.

Regarding the different nurses, senior nurses rated the highest on SP (3.91) and nurse assistants rated the highest on ND (3.57), whereas practical nurses rated the lowest on NVL (1.92). It indicated that senior nurses perceived more importance of fatigue from SP than others, whereas nurse assistants perceived the most importance on ND, which was in the accordance with the results of the physical fatigue (Figure 7).

## Discussion

### The Primary Environmental Design Factors Relevant to Nurse Fatigue in IDNU

This study demonstrated that the physical environment of IDNU could affect the fatigue of nurses, which is consistent with the previous studies on the other healthcare settings (9, 26). This study further showed that the environmental factors could be sorted into four main categories of ND, NVL, SP, and SC, which were identified as the basic parameters affecting the fatigue from the environmental design. In the nursing unit, ND is associated with the daily walking of nurses, and the larger ND is, the more distances nurses have to walk (37). ND could be affected by the plan layout, the location, and the size of the nursing station, indicating that fatigue could be interfered by the spatial design. SC and SP are thought to affect the perceptions of the nurses about the working environment. A crowded and intense working environment could make nurses nervous and stressed, especially during COVID-19. Similarly, the lack of SP could reduce the sense of security of the nurses, hindering their recovery from the heavy workload. The sense of privacy is thought to be associated with the psychological restoration spatially. NVL is supposed to consist of daylighting and fresh air, as well as the restorative effect natural resources by enhancing the perceptions of the nurses (45). Besides, it could provide nurses' sense of bio-safety and escaping from exposure in IDNU, which is often filled with the highly contagious pathogens.

These factors are thought to have more influence on the physical fatigue than the psychological fatigue, generally. The mean value of the environmental factors on the physical fatigue is all higher than that of psychological fatigue (Table 3), especially for ND (3.92). The advantage on the physical fatigue, may attribute to the surging amount of workload due to COVID-19. In another side, NVL was not found to be significantly associated with the fatigue (Table 3), which could be different from the previous studies (26, 41). The possible reason is that, in COVID-19, nurses in IDNU were always overwhelmed by work and not able to rest for long, which discourage them to benefit from restoration from natural resources. The prior studies showed that increasing the proportion of single-bed wards would reduce the fatigue and stress of nurses (9, 26, 46). Such results are not found in this case because double-bed and triple-bed wards are still the primary configuration in the nursing unit of developing countries due to limited healthcare resources. To illustrate, increasing the proportion of single-bed wards would barely alleviate the fatigue because it would increase the nursing distance by enlarging the size of nursing unit. Anyhow, this study reveals the dilemma of the hospitals in the developing countries, where there is a large demand of healthcare service but limited resources. These studies may be different from the studies in the developed countries.

### Nursing Distance and Spatial Privacy as the Main Environmental Design Factors

Nurses did not consider these environmental factors equally. ND and SP were thought to be the principal factors that influence the physical and psychological fatigue, respectively (Table 3). In accordance to the previous study, ND is found to be the main factor on the physical fatigue because it is closely associated with the daily walking distance for nurses. Cruising between patient rooms and nursing station is a large stressor for the work-related fatigue (6). For a routine, nurses need to walk on almost every step of their work from reading the instructions of doctors at the nurse station, ensuring the instructions in the wards, and entering the records after returning to the nurse station. The increasing walking distance due to COVID-19 makes nurses more likely to be physically exhausted than normal. Hence, ND is supposed to be the main factor on the physical fatigue.

SP is thought to be the most prominent factor on the psychological fatigue. The previous studies paid more attention on the patients than nurses regarding the perceptions of privacy (26). This study showed that the perceptions of nurses on the fatigue could also be affected by the sense of spatial privacy, especially for low-ranking nurses. In China, nurse assistants and practical nurses often have to share their working and resting space with others, leading to the lack of privacy. The possible reason could be the limited spatial resources of nursing unit, and the priority is always given to the needs of patients, doctors for the medical function. There is little considerations of privacy on the low-ranking nurse, as a result, they could suffer from the fatigue due to the disruptions such as noise and wrong way finding (9). Moreover, increasing SP by separating nurses away from the public could enhance their sense of safety. Owing to frequent incidents of assaulting healthcare workers in China, nurses would feel safe if their private areas are ensured with restricted access. Therefore, for the sake of their safety and well-being, the issue of privacy should be improved by environmental design such as setting up private break areas, common space, and restricted access.

### Comparison of Physical and Psychological Fatigue by Environmental Factors

Nurses perceived the distinct influence of environmental factors on the fatigue. To clarify, a stronger influence on the physical fatigue than the psychological fatigue was found in this study, particularly for practical nurses and nurse assistants. Normally, they are more physically involved with the daily workload. It could also be proved by the ratings of ND, which was scored the highest on the physical fatigue by them. The possible reason is that COVID-19 makes the workload increase dramatically, and nurses have to walk more distances than normal, leading to the prevalence of physical exhaustion. Besides, the lifting and supporting activities during transferring patient could also contribute to the physical fatigue as the work becomes heavier. Report showed that the workload of nurses increased almost 1.5–2.0 times during the pandemic in some developing countries (14). Moreover, IDNU requires a complicated and rigorous infection-control protocol. There are two sanitary passes between three zones where they need to change gears (Figure 3). It could be very physically exhausting for nurses because they have to commute to wards for many times a day. Such studies are not evident for the senior nurses as they are less physically involved. Senior nurses are more vulnerable to psychological fatigue as their jobs are more about the management and instruction.

### The Role of Different Nurses

Technical titles showed significant differences for nurses on their perceptions of the environmental factors. Regarding the physical fatigue, practical nurse, and nurse assistant rated higher than senior nurses did. It indicates that low-ranking nurses are more likely to be affected by these environmental factors on the physical fatigue. In IDNU, the work of low-ranking nurses is more physically oriented than senior nurses. In the pandemic, they walked longer than normal, and would not stay in one place for long because they needed to keep commuting to ensure the condition of the patients of COVID-19. As a result, they were more vulnerable to physical fatigue than senior nurses. As the low-ranking nurses account for the majority of the nurse staff (87.5%) in this case, it highlights the demand to put more attention on the low-ranking nurses when conducting environmental interventions.

Senior nurses were found to be more affected on the psychological fatigue by these environmental factors, which aligned with the previous studies that they were more likely to suffer from the psychological fatigue. Their daily work is mainly about management, instruction, and supervision; and their frequent working places are often at the nursing station and office (Figure 5). The characteristics of their work are less physical exhausting but could produce more stress. As a result, their walking range is not as large as low-ranking nurses, and they are less affected by the physical fatigue than the psychological fatigue by the environment.

### Implications to the Practice of IDNU by Environmental Design

Environmental interventions and strategies should be presented to help designers and decision-makers to improve the working environment of frontline nurses. According to the findings of main environmental factors on the fatigue, the design recommendations on ND and SP would be emphasized.

Reducing ND could be the primary design advice. It is suggested to locate the nurse station in the middle of IDNU instead of in the end to reduce the maximum nursing distance for one direction. There are two types of IDNU plan: middle-mode and end-mode, depending on the location of the nursing station. The middle-mode could be more beneficial to nurses fatigue because the distance to wards would be balanced for both directions. For the large IDNU, setting up a secondary nursing station could be a solution to reduce the nursing distance (37).

As for SP, creating an independent break area for nurses could be beneficial to alleviate the fatigue. The design measures could also intensify this effect such as arranging the patio and balcony with access to nature. It could help nurses recover from the heavy workload and other disturbances. Improving privacy by setting up the independent areas could also promote their sense of safety by separating them from patients and visitors. With the access to nature, it could further provide nurses restoration and sense of escaping from the stress.

### Limitation

As a survey and interview-based study, it is limited in the sample size. Nonetheless, the IDNU of this study is representative for its environmental features and nurses, one hospital could limit the scope of the findings and dynamic behind it. After the restrictions of IDNU are removed as COVID-19 gradually under control, IDNU from more hospitals of different levels could be explored to find out the dynamic behind the environment and fatigue. In the future, with the development of wearable devices, mobile app and online instruments, more objective and detailed information could be collected.

## Conclusion

Reducing the fatigue of frontline nurses in IDNU is a prominent issue to address during COVID-19. IDNU plays an important role in fighting against this highly contagious disease in the developing countries. This study applied a mixed method to analyze the environment and the fatigue of nurses from physical design. The results suggest that the fatigue could be affected by environmental factors such as ND, SP, NVL, and SC. These factors are found to be more influential in the physical fatigue than psychological fatigue. ND is found to be the main factor in the physical fatigue, while SP is in the psychological fatigue. The technical titles could affect the perceptions of nurses on the fatigue by these environmental factors. Low-ranking nurses are more likely to suffer from physical fatigue than high-ranking nurses, who are mainly affected by the psychological fatigue. Environmental interventions such as reducing the nursing distance and improving the privacy of nurses by design are prompted to alleviate the fatigue. However, there is no one-size-fits-all blueprint due to their different conditions, and it is necessary to take adaptive interventions. Overall, this study provides preliminary evidence to the healthcare design and nursing management, in identifying environmental factors that could be utilized to mitigate the fatigue of nurses. The findings are expected to foster the safety and well-being of nurses, and allow them to deliver better service in the COVID-19.

## Data Availability Statement

The data could be available upon reasonable requirements through corresponding author due to the agreement with participants.

## Ethics Statement

Ethical review and approval were not required for the study on human participants in accordance with the local legislation and institutional requirements. Written informed consents to participate in this study were provided by the participants either online (nurses) or in the written form (experts).

## Author Contributions

MM and MA made substantial contributions to the conception, design, analysis, and interpretation of data. HL made substantial contributions to the acquisition and analysis of data. BH revised the final manuscript. MM was responsible for the funding acquisition. All authors were involved in drafting and critically revising the manuscript for intellectual content. All authors have agreed to be accountable for all aspects of the work and have read and approved the final manuscript.

## Funding

This research was funded by the Natural Science Foundation of China (Grant No. 5200081561) and Fellowship of China Postdoctoral Science Foundation (Grant No. 2021M693729).

## Conflict of Interest

The authors declare that the research was conducted in the absence of any commercial or financial relationships that could be construed as a potential conflict of interest.

## Publisher's Note

All claims expressed in this article are solely those of the authors and do not necessarily represent those of their affiliated organizations, or those of the publisher, the editors and the reviewers. Any product that may be evaluated in this article, or claim that may be made by its manufacturer, is not guaranteed or endorsed by the publisher.

## Appendix

### Questionnaire for Nurses' Opinions of the Environmental Factors on Physical and Psychological Fatigue

**Table:**

Part 1. Individual Information				
Technical titles	Nurse assistant	Practical nurse	Senior nurse	Mark the options if applicable
Age				
Gender				
Education Level	Junior college and below	Undergraduate	Master degree and above	
Length of profession	1–4 years	4–9 years	>10 years	
Work duration in COVID-19 (full time)	<25 days	More than 25 days (including 25 days)		

**Table:**

Part 2. Environmental factors of IDNU and fatigue			
Instruction: how do you feel about the environmental factors affect your physical fatigue in the IDNU? Please rate from 1 to 5, 1 (not at all or little), 5 (very much or quite)			
	Physical fatigue	Psychological fatigue
A Nursing distance	A1 Plan layout	
	A2 Location of nurse station	
	A3 Number of wards	
B Spatial crowdness	B1 Size of nurse station	
	B2 Ratio of supportive rooms	
	B3 Width of corridor	
C Natural light and ventilation	C1 If windows of natural light in the nurse station	
	C2 If openable windows in the end of corridor	
	C3 If access to natural light and ventilation in the break area	
D Spatial privacy	D1 Independent break area for nurse	
	D2 Safety of workplace	
	D3 Typology of nurse station (open/semi-open/closed)	

**Table:**

Part 3. The locations you frequently stay and work in the IDNU You can mark on the plan we provide. Every participant allows to mark three points at most.
